# Influence of Ca^2+^ on Early Degradation of Cast-In-Situ Mortar Induced by Sulfate-Magnesium Multiple Combined Attack

**DOI:** 10.3390/ma15165752

**Published:** 2022-08-20

**Authors:** Gaowen Zhao, Mengzhen Guo, Shaomin Li, Xiaolin Weng, Shijun Ding, Fangzhou Han, Haoran Wang

**Affiliations:** 1 School of Highway, Chang’an University, Xi’an 710064, China; 2 Key Laboratory for Special Area Highway Engineering, Ministry of Education, Chang’an University, Xi’an 710064, China; 3 China Electric Power Research Institute, Beijing 100085, China

**Keywords:** cast-in-situ mortar, internal magnesium attack, internal–external sulfate attack, Ca^2+^

## Abstract

Early degradation of cast-in-situ concrete induced by multiple internal–external sulfate combined attacks significantly affects the development of concrete strength. An experimental study regarding the effects of Ca^2+^ on the early degradation of cast-in-situ mortars subjected to internal–external sulfate and magnesium combined attacks is investigated in this paper. In particular, a specific method for accurately simulating the degradation of cast-in-situ structures was proposed in this experiment. Physical properties (including weight, size changes, and porosity), mechanical properties (including flexural strength and compressive strength), sulfate concentration, and microstructural properties were monitored during 28 days of immersion. The results show that an internal sulfate and magnesium combined attack (ISA-IMA) obviously retards the development of early strength and accelerates the degradation induced by external sulfate attack (ESA). The diffusion path of sulfate ions from outside is blocked by flake-shaped magnesium hydrates, delaying the penetration of external sulfate attacks. However, it is far from neutralizing the strength loss induced by an internal magnesium attack (IMA) at an early age. Premixed excessive Ca^2+^ would improve the strength development and pore structure of concrete or mortar, enhancing durability against corrosive conditions.

## 1. Introduction

Degradation damage induced by sulfate attack has been extensively studied in recent years. It always occurs in cast-in-situ concrete structures such as bridge foundations, piles and tunnels. Sulfate attack can be divided into three types on the basis of sulfate sources: external sulfate attack (ESA), internal sulfate attack (ISA) and internal–external sulfate combined attack (CSA) [1].

At present, there is abundant research on ESA and its corresponding degradation mechanisms. ESA is a complex process that involves the transfer of sulfate ions through the pores (diffusion, permeation and capillary adsorption) and the corrosion reactions of aggressive ions with cement composites [2]. Corrosion products of sulfate attack mainly consist of ettringite and gypsum, which can be induced by ESA, ISA and CSA. Ettringite is also a type of normal hydration product in cement paste (reaction products by aluminates, sulfates and calcium hydroxide). Its generation only becomes harmful when it occurs after the hardening process of cement in the hardened rigid cement matrix [3]. Expansive corrosion products cause the degradation and strength loss of concrete.

It is well documented that ISA in engineering constructions such as tunnels, pavements, pile foundations and bridges would generate delayed ettringite and decrease the service life of these structures [4,5]. ISA occurs in concrete structures due to the presence of mixed sulfates in aggregates and raw materials [6,7], or the use of sulfate-contaminated water during the mixing process. ISA is considered to be a degradation process that produces expansive secondary ettringite and causes damage [8]. The properties and durability of concrete are negatively affected owing to the dimensional variation, increase in internal stresses, and cracks [9,10]. In this regard, internal sulfates would cause degradation soon since they need no diffusion process [3]. Previous studies have shown that premixed sulfates promote the degree of hydration first and then retard the hydration degree of cement in the early stage. Therefore, ISA has an adverse impact on the early strength of concrete [11]. Fu found that mortars made with cement that contained high sulfate content exhibited higher expansion after 14 days [12]. Zhao conducted an experiment on cast-in-situ mortar and found that ISA would accelerate the degradation process and the development of cracks and expansion in a short time [13].

Except for ISA, the effects of other internal corrosive ions cannot be ignored. In saline areas, the use of water and aggregates containing multiple corrosion ions (such as SO_4_^2−^, Mg^2+^, Ca^2+^) would cause serious internally induced degradation (such as an internal magnesium attack, IMA). Moreover, the degradation induced by environmental magnesium salts is the most serious of these aggressive ions [14,15]. However, evidence shows that magnesium blocks the paths that help sulfate ions penetrate internal concrete, subsequently retarding the corrosion of sulfates in sulfate-rich environments [16]. Magnesium hydroxide (corrosion products of magnesium sulfate and calcium hydroxide) forms a protective layer on the concrete surface, reducing ionic transport at an early stage until it is mechanically damaged [17]. Higgins found that the strength of specimens immersed in magnesium sulfate was higher than those immersed in sodium sulfate after a long soak [18]. In addition, Williams thought that magnesium is aggressive, while sodium and calcium are uninjurious [19]. Neville found that calcium sulfate would cause the formation of ettringite but not always lead to expansion [20]. Studies have shown that the calcium needed for mono-sulfate to ettringite conversion is provided by calcium sulfate; no additional calcium originating from calcium hydroxide or the C-S-H phase is needed. This would avoid the decalcification of the C-S-H gel and enhance the strength of cement or mortar [6]. Nevertheless, in recent years, the degradation mechanisms of cast-in-situ concrete suffering from IMA and the effects of Ca^2+^ on concrete subjected to ESA-ISA-IMA have rarely been reported.

At present, studies on sulfate attacks have focused more on precast concrete structures than on cast-in-situ structures. In fact, cast-in-situ concrete is widely used to improve the integrity and aseismic of structures. Internal–external corrosion reactions would proceed once cast-in-situ concretes are put into environments. Therefore, the degradation mechanisms between precast and cast-in-situ concrete are not the same. Based on the aforementioned research, the effect of Ca^2+^ on cast-in-situ mortar degradation induced by an internal–external sulfate combined magnesium attack (ESA-ISA-IMA) in the early stage was studied in this paper. An organic glass mold was designed and used to simulate the degradation of cast-in-situ mortar subjected to ESA during early hydration. The cement mortar specimens were cast and immersed in 7% sodium sulfate solution and distilled water, respectively. The effects of Ca^2+^ and ISA-IMA were simulated by adding 3% (mass ratio) calcium, sulfate or magnesium salts in mixing water. Apparent changes, physical properties (weight, size and porosity), and mechanical properties of mortar specimens were determined at different immersion times. The sulfate concentration at different depths from the exposed surface was measured using chemical titration. Microstructural and mineral analyses were conducted in succession by scanning electron microscopy with energy dispersive spectroscopy (SEM-EDS), X-ray diffraction (XRD), and thermogravimetric analysis (TG/DTG).

## 2. Experimental Materials and Methods

### 2.1. Materials

This study used Portland cement (P.O. 42.5, made in Shandong China) and standard sand (made in XIAMEN ISO Standard Co., Ltd., Xiamen, China) with a size range of 0.08–2 mm to prepare specimens. Distilled water was used to prepare both the specimens and the solutions. AR Na_2_SO_4_, Mg(NO_3_)_2_, Ca(NO_3_)_2_ produced by Xilong Scientific Chemical Industry were used in the present study. The content of the chemical composition of the cement is listed in Table 1. The w/c ratio and mix proportion of the cement mortars are listed in Table 2.

### 2.2. Specimens and Immersion Solution Preparation

Two kinds of specimens were prepared in this study and their specific purposes are listed in Table 3. Two kinds of solutions were prepared: 7% sodium sulfate solution and distilled water. Internal corrosive sources were simulated by premixing the corresponding salts into distilled water, which was used to prepare the specimens. Detailed information on the specimens is illustrated in Table 4.

**Figure 1:**
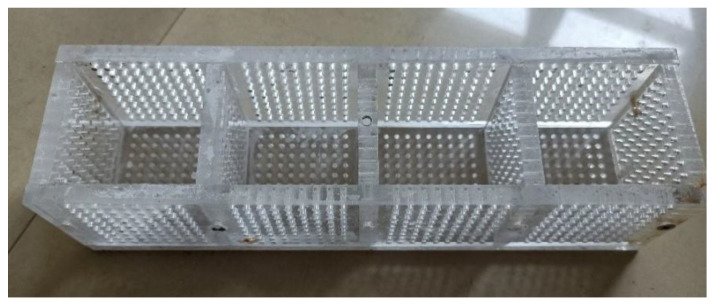
Organic glass molds with through-holes.

To simulate the situation in which cast-in-situ mortars are immediately exposed to sulfate corrosion environments after being cast, an organic glass mold with through-holes was used in this experiment (as shown in Figure 1). These 2 mm through-holes in the mold allowed external sulfate ions to diffuse inside mortar before molds were dismantled. The proposed method could investigate the early degradation of cast-in-situ structures that suffered ESA. This method could simulate the degradation process before the entire hardening of concrete or mortar, thus making the experimental results closer to actual engineering. In addition, glass paper and filter papers were attached inside molds to prevent the mortar mixture from blocking these through-holes.

Cement and water were mixed for 30 s, and then standard sand was added to the mixer and mixed for 60 s. After 60 s, it continued to rapidly mix for 60 s. Then, the mortar mixture was poured into an organic glass mold. After 10–15 s vibration, glass paper was removed and specimens with molds were full-immersed in distilled water or sulfate solution, respectively. The liquid level of the solution was always maintained 3 cm higher than that of the mortar specimens. After 24 h, the molds were dismantled, and filter papers were also peeled. These specimens were removed from the solutions at 1, 7, 14 and 28 days. A temperature of 23 ± 2 °C and a relative humidity (RH) of 65% were maintained during the whole experiment.

### 2.3. Test Methods

#### 2.3.1. Physical and Mechanical Properties

Changes in weight and size of specimens were measured by a balance and Vernier caliper, with an accuracy of 0.1 mm and 0.01 g, respectively. The weight of the specimens was measured twice to obtain an average value. The size of the specimens was measured five times the length of the specimens. Flexural strength of the specimens was determined by a DKZ-5000 electric type flexural testing machine, with a loading rate of 3 kN/min. Compressive strength was measured by a multi-functional (the model is POW) loading system, with a loading rate of 5 kN/min. MIP test (the model is YG-97) was performed to detect the pore distribution of mortars suffering from different corrosion conditions. The sizes of the samples used to conduct the MIP test were 20 mm × 20 mm × 20 mm.

#### 2.3.2. Sulfate Concentration

Specimens were taken from the sulfate solutions and distilled water after being immersed for 1, 7, 14 and 28 days. These specimens were then put into the drying oven at 60 °C for 48 h. A percussion drilling was used to collect 5 powder samples every other 5 mm beneath the exposed surface of specimens. 0.5 g powder was dissolved in 50 mL distilled water to dissolve all sulfates. After 48 h, chemical titration was used to measure the sulfate concentrations of the specimens.

#### 2.3.3. SEM-EDS, XRD and TG/DTG Analysis

Powder samples were obtained by drilling into internal mortar at a depth of 5 mm to perform XRD and TG/DTG analysis. Samples used to take SEM images and EDS analysis were drilled into core samples. The ZEISS-SIGMA 300 system was used to observe the microstructure images and the EDS analysis. XRD test was conducted with the model RINT 2000 to determine the mineral composition of mortars with a voltage of 40 kV. The scanning speed and current during the test were set as 5°/min and 100 mA. The temperature during the TG/DTG (Model NETZECH-STA 449C system) analysis increased from 30 to 1000 °C in a nitrogen atmosphere and the heat rate was 20 °C/min.

## 3. Results

### 3.1. Changes in Weight, Size and Appearance

#### 3.1.1. Weight Change

**Figure 2:**
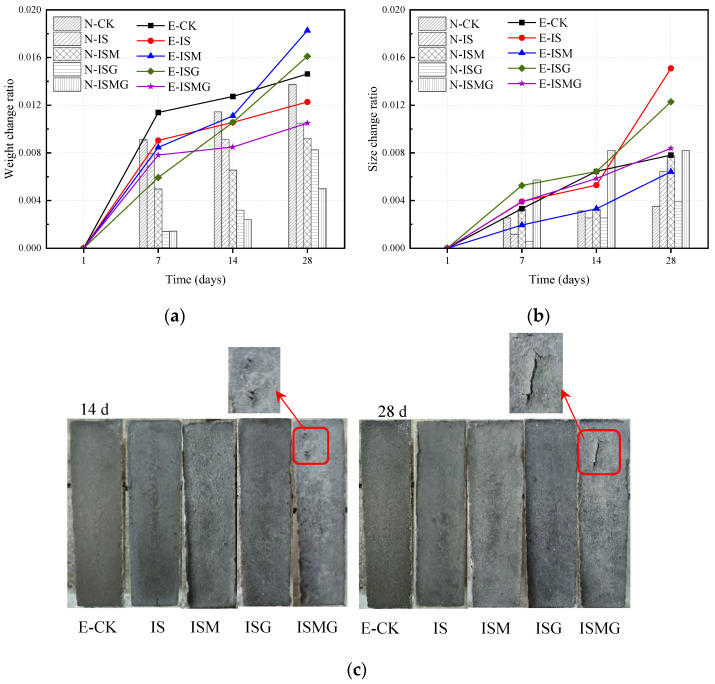
Change of specimens in weight, size and appearance under different corrosion conditions during different periods: (**a**) weight change, (**b**) size change, (**c**) appearance of mortars exposed to external sulfate solution.

The change in specimens’ weight and size is a general index to reflect the deterioration degree of concrete or mortar exposed to sulfate environments. Figure 2a shows the weight change ratio of mortars, obtained by (*M_t_* − *M*_1_) × 100%/*M*_1_ (in which *M_t_*, *M*_1_ represent the weight of specimens immersed for *t* day and 1 day, respectively). The bar graph represents the weight change ratio of specimens without suffering ESA and the solid line represents the weight change ratio of specimens exposed to sulfate solution.

As can be seen in Figure 2a, the weights of EC (EC represents the specimens exposed to sulfate solution) show a more significant change in comparison to NC (NC means the specimens immersed in distilled water) during the early stage. This is because external sulfate ions penetrate mortar and react with cement hydration products or cement components, generating corrosion products, such as ettringite and gypsum. These corrosion products usually load great expansion pressure on internal specimen cracks to enhance the density of the specimen. Specimen E-ISG had a considerable weight change compared to specimen E-IS after 28 days of immersion. Specimen E-ISM presents the fastest growing trend during the corrosion process. While the weight of specimen E-ISMG quickly increases in the first week, the growth decelerates until the end of immersion.

Early weight changes always relate to cement hydration and reactions between corrosion ions and cement composites. For specimens that suffered ISA-IMA, degradation is thought to be caused by the formation of ettringite, magnesium hydrates and M-S-H, which is a non-cementitious phase generated after the magnesium-induced attack [21]. Expansive corrosion products and magnesium hydrates destroy the hydrated cementitious matrix and cause mass loss in specimens [22]. Thus, weight-change ratios present a lower level for specimens that suffered from ISA and ISA-IMA. For specimens with premixed calcium salts, excessive Ca^2+^ would influence the hydration process by accelerating the hydration degree of C_3_A [6]. The generation of abundant hydration products would also contribute to the increment in weight in the early stages.

#### 3.1.2. Size and Appearance Changes

Figure 2b shows the size changes of specimens immersed in the sulfate solution and distilled water for 1, 7, 14 and 28 days. Obviously, the size change ratio of EC remains higher than that of NC because of the accumulation of corrosion products in the mortar surface layer.

As shown in the curve of E-IS, CSA causes significant expansion owing to the formation of abundant ettringite. The size change ratio of specimen E-ISG reduces by 27% compared to E-IS at 28 days. For specimens E-ISM and E-ISMG, sizes show a little change during the early immersion. This is due to the fact that magnesium attacks are characterized by the loss of mortar strength rather than expansion [6]. For NC specimens, the size change is mainly related to cement hydration and ISA-IMA. Attributing to the lack of ESA, the change ratios of specimens N-IS and N-ISG are distinctly lower than corresponding EC specimens (E-IS and E-ISG). It can also be seen that Ca^2+^ slightly restricts the expansion induced by ISA by comparing N-IS and N-ISG. This can be ascribed to the fact that premixed excessive Ca^2+^ promotes cement hydration since Ca^2+^ is a vital reactant in cement hydration [13]. Such a process would restrain the reactions between cement composites and SO_4_^2−^, and decrease the expansion induced by corrosion products at early hydration. For specimen N-ISMG, the size change ratio is the highest among all NC specimens. This indicates that internal–external multiple ion attacks produce more corrosion products inside cement mortar.

Figure 2c shows the appearance changes for mortars that suffered ESA at 14 and 28 days. It is clearly indicated that ESA-ISA-IMA would not cause visible appearance differences for cement mortars in the early stage, except for specimen E-ISMG. Peeling can be observed on the specimen E-ISMG surface (shown in Figure 2c). In sulfate-rich environments, sulfate ions permeate into the interior mortar through capillary effect before hardening and taking part in the hydration process. Such a process would generate and accumulate expansive corrosion products in the surface layer of the mortar. At the same time, internal Ca^2+^, Mg^2+^ and SO_4_^2−^ would participate in the process of both hydration and corrosion. Abundant hydration products and corrosion products are gathered in the mortar matrix. Thus, peeling and cracks occur on the surface of the mortar.

### 3.2. Mechanical Properties

#### 3.2.1. Flexural Strength

**Figure 3:**
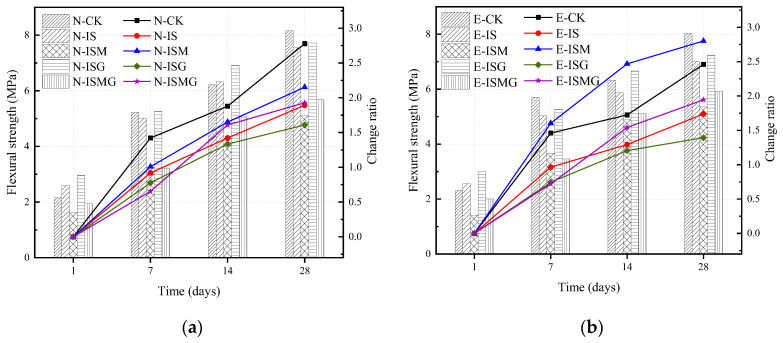
Flexural strength and change ratio of specimens in different stages: (**a**) NC specimens and (**b**) EC specimens.

Figure 3a,b show the flexural strength and change ratio for specimens immersed in distilled water and sulfate solution, respectively. The change ratio was calculated by (*f_t_* − *f*_1_)/*f*_1_, in which *f_t_* and *f*_1_ represent the strength of the specimen at *t* days and 1 day, respectively.

For EC specimens, the flexural strength of E-IS is lower than that of E-CK. After premixing 3% calcium salts (E-ISG), the strength increments after immersing for 1, 7, 14 and 28 days are 0.46, 0.24, 0.79, and 0.22 MPa, respectively. In addition, specimens that suffered IMA (E-ISM and E-ISMG) show a higher loss of strength compared to those that suffered no IMA. In addition, for specimen E-ISMG, the flexural strength increments at 1, 7, 14 and 28 days are 0.6, −0.21, 0.22, and 0.56 MPa compared to specimen E-ISM.

#### 3.2.2. Compressive Strength

**Figure 4:**
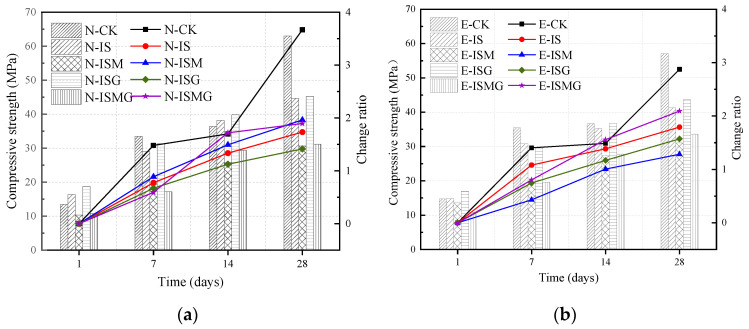
Compressive strength and change ratio of specimens in different stages: (**a**) NC specimens and (**b**) EC specimens.

Figure 4a,b shows the compressive strength and growth ratios of specimens exposed to distilled water and sulfate solution, respectively. The results indicate that the strength of all specimens demonstrates an incremental trend at an early stage. The strength of NC grows faster than that of EC.

For EC specimens, as shown in Figure 4a, the compressive strength of specimen E-CK rapidly grows at the early stage. This could be explained by the continuous hydration of cement composites condensing the microstructure of mortar [23]. The growth rates of the other specimens obviously decrease after 14 days, except for specimen E-CK. In addition, the strength of specimens that suffered IMA is lower than those without IMA. The compressive strength of specimen E-ISM at 1, 7, 14 and 28 days decreases by 7.7%, 36.8%, 22.8%, and 24.8%, respectively, compared to specimen E-IS. For specimens with premixed extra Ca^2+^, the compressive strengths of specimens E-ISG and E-ISMG are 12.8%, 3.8%, 3.9%, 5.2% and −2%, 1.7%, 1.6% and 7% higher than specimens E-IS and E-ISM, respectively, after immersing for 1, 7, 14 and 28 days.

It can be seen from the results of flexural strength and compressive strength that whether cast-in-situ mortars suffer internal–external multiple attacks or not, strength would always be ever-increasing at the early stage. ESA would obviously retard the early increment of strength since cast-in-situ mortar would suffer sulfate attack once is poured into sulfate-rich environments. ISA would expedite early damage to mortar induced by ESA due to the generation of more degradation products. Furthermore, the corrosion products generated by ISA-IMA remarkedly restrain the growth of strength in the early stages. However, for specimens with premixed calcium salts, Ca^2+^ would participate in the hydration process and promotes the formation of hydration products. Many hydration products fill the pores of mortar and make the pore structures denser. In this regard, Ca^2+^ premixed in mortar increases the strength of mortars suffering from ISA or ISA-IMA during the early stage.

### 3.3. Porosity

Porosity and pore size distribution are significant indexes that affect the mechanical properties and durability characteristics of hardened concrete or mortar [24]. The gel pore, usually less than 0.01 μm, would not adversely influence the strength of the mortar. However, capillary pores and other pores, which are mostly over 0.01 μm, are responsible for the reduction of strength and durability [25]. Therefore, in this paper, the pore size of mortar is generally divided into small pore (0.01–0.1 μm), middle pore (0.1–10 μm) and big pore (>10 μm).

**Figure 5:**
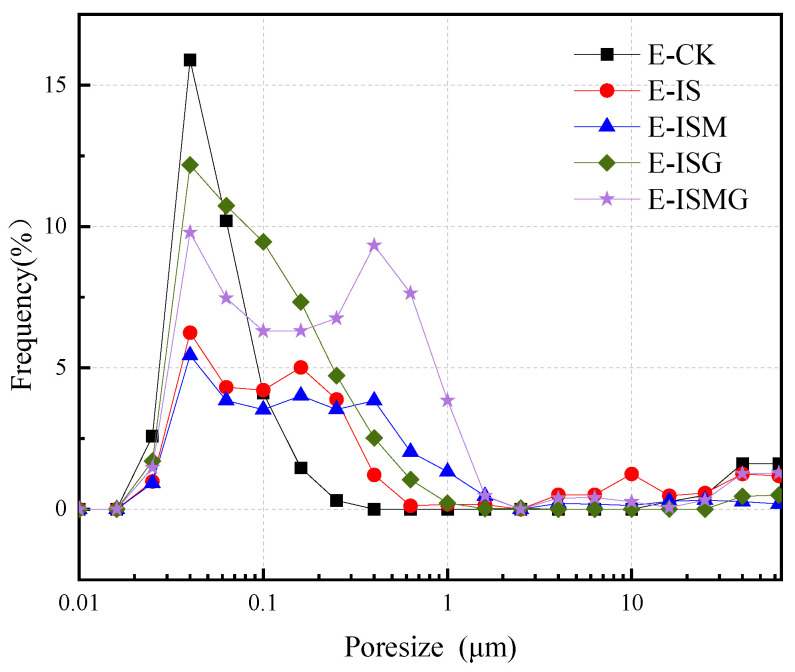
Distribution of pores for EC specimens at 28 days.

Figure 5 shows the distribution of the pore radius for the EC specimens after 28 days of immersion. For specimens E-IS and E-ISM, the proportions of small and middle pores are lower than that of specimen E-CK. The corrosion products produced by sulfates and magnesium salts fill small pores and middle pores during the early process of hydration. Small and middle pores and cracks would be further expanded due to the accumulation of expansive corrosion products. In addition, it can be seen clearly that the proportions of small pores and middle pores are extremely increased owing to the existence of calcium salts. Premixed excessive Ca^2+^ would contribute to early hydration and produces more hydration products. This would improve the pore structure of the mortar, decreasing the damage induced by larger pores. Further, the function of Ca^2+^ on improving the proportions of small pore and middle pore would increase the durability against ESA.

### 3.4. Sulfate Concentration

**Figure 6:**
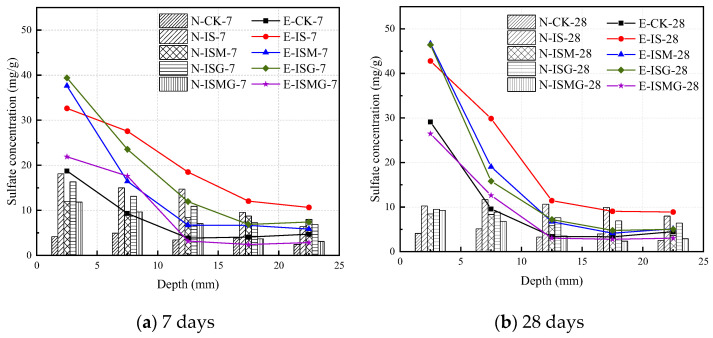
The sulfate concentration for specimens immersed in sulfate solution and distilled water at 7 days and 28 days, respectively.

Figure 6 shows the changes in sulfate concentration at different depths against the immersion time. Figure 6a,b represents the sulfate concentration of the specimens after immersing for 7 days and 28 days, respectively.

For NC specimens, sufficient corrosion ions join in the hardening process and react with cement composites in the early stage to generate corrosion products. Figure 6 shows that the concentration of dissociative sulfate ions decreases gradually as soaking time grows. For EC specimens, the diffusion speed of sulfate ions is rapid at the early stage since cast-in-situ mortar would suffer ESA immediately when it is put into sulfate solution. Sulfate concentration increases over immersion time while reducing with the increase of depth beneath the exposed surface. The sulfate content in the surface layer is universally higher for mortar specimens subjected to ISA-IMA.

Environmental sulfate ions permeate into mortar through pores, reacting with cement hydration products to cause degradation. In addition, internal sulfate and magnesium ions react with cement composites, and then plenty of expansive degradation products are gathered in the pores of the mortar. The crystallization pressure induced by these products on the pore walls exceeds the tensile strength of mortar [26]. Soon later, the formation of microcracks would be observed. Microcracks on the surface of the mortar speed up the diffusion of external ions. For specimens premixed excessive Ca^2+^, the sulfate content is significantly lower than those without premixed Ca^2+^. This is because Ca^2+^ takes part in the process of hydration and corrosion, contributing to the hydration of cement and the consumption of sulfate ions at the same time. Therefore, more hydration products and corrosion products inside the mortar compact the mortar structure and improve the early strength.

### 3.5. Microstructure and Mineral Analysis

**Figure 7:**
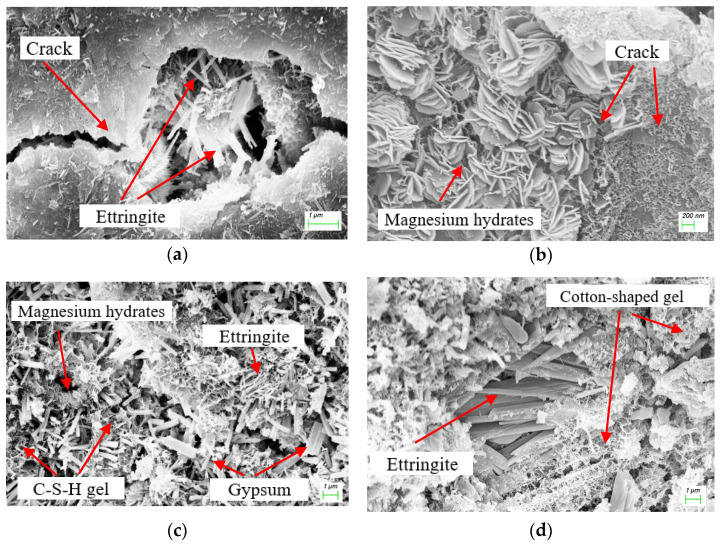
SEM results for specimens: (**a**) E-IS, (**b**) E-ISM, (**c**) E-ISMG and (**d**) E-ISMG.

**Figure 8:**
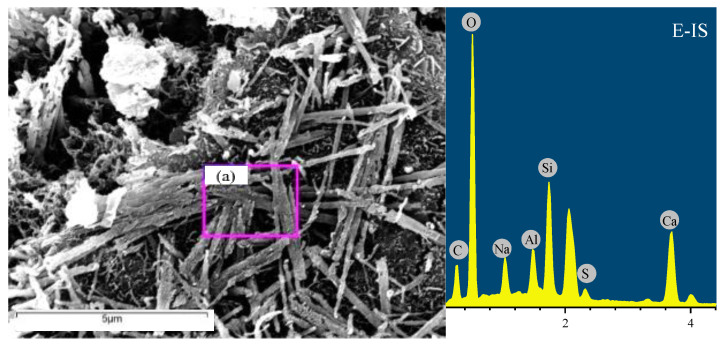

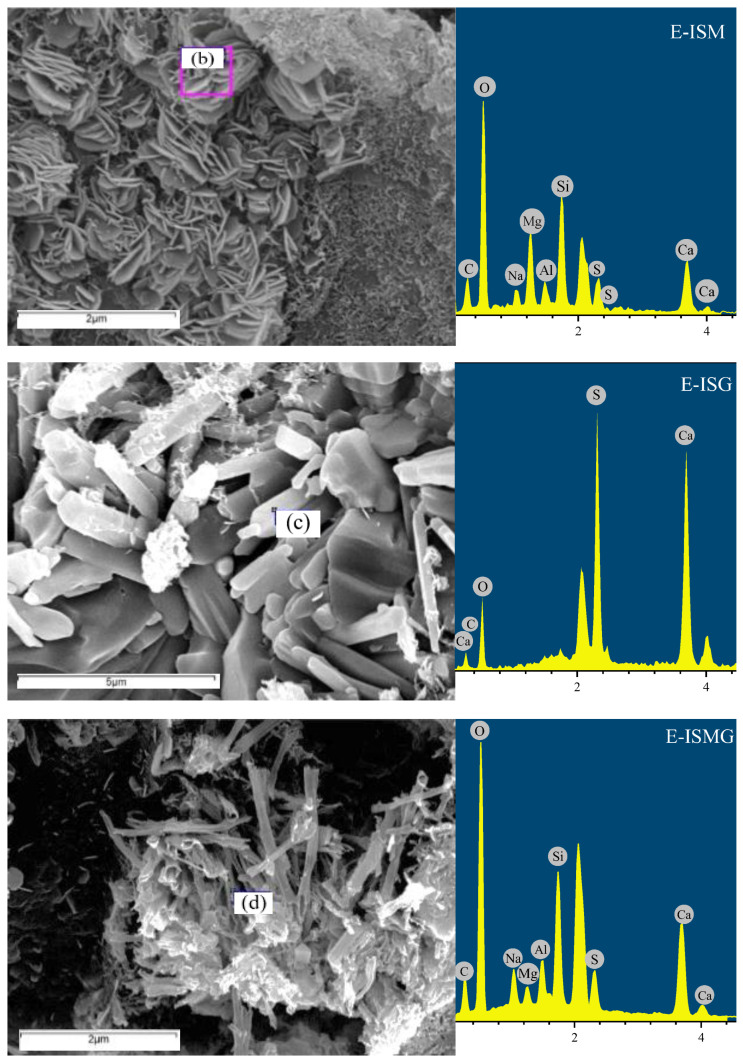
SEM observations and EDS results for specimens immersed in sulfate solution at 28 days: (**a**) E-IS, (**b**) E-ISM, (**c**) E-ISG, and (**d**) E-ISMG. Note: purple box is the selected area to do EDS analysis.

To determine the corrosion products of ESA-ISA-IMA after 28 days of immersion, SEM-EDS, XRD and TG/DTG tests were conducted in succession. Figure 7 shows SEM images of the mortar specimens. Results of EDS analysis are put on the right side of SEM images in Figure 8a–d, which represent the specimens E-IS, E-ISM, E-ISG and E-ISMG, respectively.

Images of SEM with corresponding EDS results show that ettringite and gypsum are the main corrosion products when mortar specimens suffer ESA-ISA-IMA. The pores of mortar specimens are filled with these expansive crystalized products. Finally, the crystallization pressure loaded on the pore walls causes obvious cracks and severe damage. In addition, the microstructures of magnesium hydrates are mainly in the shape of flake hexagons, which are quite different from those of ettringite and gypsum. In particular, magnesium hydrates of flake structure would grow perpendicular to microcracks, as shown in Figure 7b. Such structures would weaken the expansion of microcracks and decrease the diffusion speed of external sulfates to some extent. For specimens premixed with calcium salts, there are more C-S-H gel and cotton-shaped gel around ettringite, as shown in Figure 7c,d. These gels form special frame structures, which are advantageous to the development of the early strength of mortar specimens.

**Figure 9:**
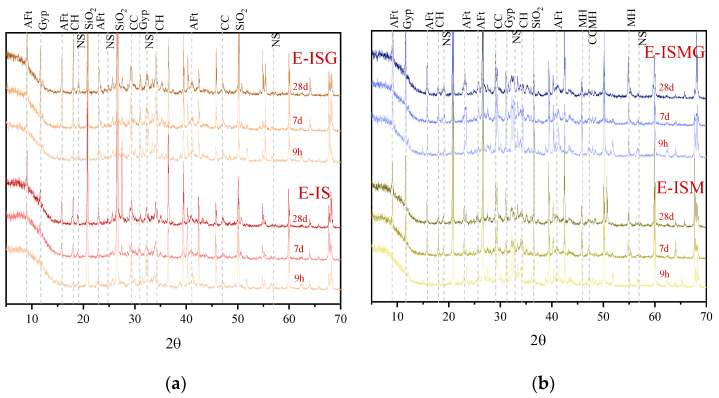
XRD results of specimens immersed in 7% sulfate solution at 9 h, 7 days and 28 days, respectively (**a**) E-IS and E-ISG, (**b**) E-ISM and E-ISMG (Aft: Ettringite Gyp: Gypsum CH: Portlandite NS: Sodium sulfate hydrate CC: CaCO_3_ MH: Magnesium hydrate).

Figure 9 shows the results of the XRD analysis of specimens immersed in sulfate solution at different periods. According to Figure 9a, the corrosion products of specimens E-IS and E-ISG mainly include ettringite, gypsum and sodium sulfate hydrates. The main chemical reactions can be listed as follows [27,28,29]:

(1) $$2{\mathrm{Na}}^{+}+{\mathrm{SO}}_{4}^{2-}+{\mathrm{xH}}_{2}O\;\Leftrightarrow \;{\mathrm{Na}}_{2}{\mathrm{SO}}_{4}\cdot {\mathrm{xH}}_{2}O$$

(2) $${\mathrm{Ca}}^{2+}+{\mathrm{SO}}_{4}^{2-}+2H_{2}O\;\to \;{\mathrm{CaSO}}_{4}\cdot 2H_{2}O$$

Ca(OH)_2_ + Na_2_SO_4_ + 2H_2_O → CaSO_4_ ∙ 2H_2_O + 2NaOH (3)

3CaO ∙ Al_2_O_3_ + 3(CaSO_4_ ∙ 2H_2_O) + 26H_2_O → 3CaO ∙ Al_2_O_3_∙ 3CaSO_4_ ∙ 32H_2_O (4)

3CaO ∙ Al_2_O_3_ ∙ CaSO_4_ ∙ 12H_2_O + 2(CaSO_4_ ∙ 2H_2_O) + 16H_2_O → 3CaO ∙ Al_2_O_3_ ∙ 3CaSO_4_ ∙ 32H_2_O (5)

For specimens that suffered IMA, there are more magnesium hydrates besides ettringite and gypsum. It should be noted that the M-S-H gel was not detected by the XRD test because of its poor crystallinity [30]. The reaction formulations are shown as follows [31]:

(6) $${\mathrm{Mg}}^{2+}+{\mathrm{SO}}_{4}^{2-}+\mathrm{Ca}(\mathrm{OH}{)}_{2}+2H_{2}O\;\to \;\mathrm{Mg}(\mathrm{OH}{)}_{2}+{\mathrm{CaSO}}_{4}\cdot 2H_{2}O$$

(7) $$3\mathrm{CaO}\cdot 2{\mathrm{SiO}}_{2}\cdot 3H_{2}O+3{\mathrm{Mg}}^{2+}+3{\mathrm{SO}}_{4}^{2-}+{\mathrm{xH}}_{2}O\;\to \;3{\mathrm{CaSO}}_{4}\cdot 2H_{2}O+3\mathrm{Mg}(\mathrm{OH}){}_{2}+2{\mathrm{SiO}}_{2}\cdot {\mathrm{xH}}_{2}O$$

2Mg(OH)_2_ + 2SiO_2_·xH_2_O → 2(MgO·SiO_2_·H_2_O) + xH_2_O (8)

**Figure 10:**
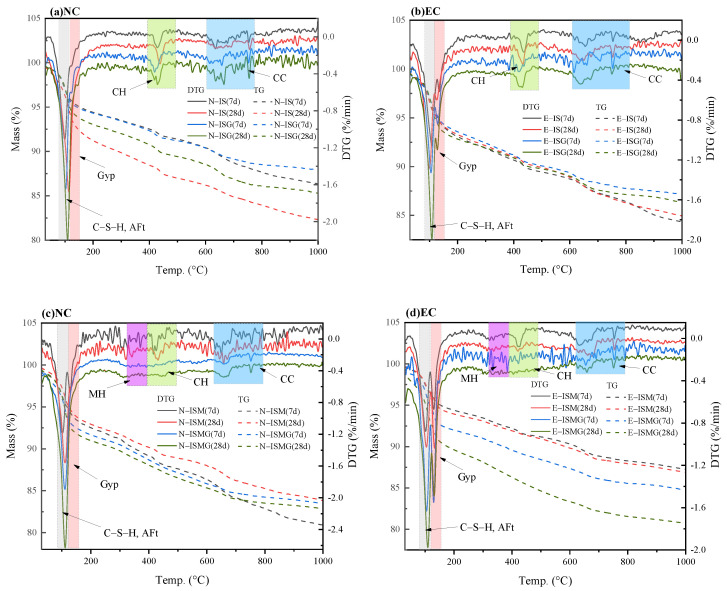
Results of TG/DTG for specimens immersed in distilled water and sulfate solution for 7 and 28 days: (**a**) N-IS and N-ISG, (**b**) E-IS and E-ISG, (**c**) N-ISM and N-ISMG, and (**d**) E-ISM and E-ISMG.

Figure 10 presents the TG/DTG curves of the degradation products for specimens exposed to sulfate solution and distilled water at 7 days and 28 days, respectively. Figure 10a,c represents the results of the NC specimens, and Figure 10b,d represents the results of the EC specimens. TG/DTG analysis results were determined by published literature [32,33,34,35,36].

The results of TG/DTG tests show that specimen EC would generate more ettringite and gypsum compared to specimen NC. Microcracks and expansion would be generated due to excessive ettringite inside the mortar. In addition, there are more magnesium hydrates, such as Mg(OH)_2_ for specimens that suffered IMA. Mg(OH)_2_ with a larger molar volume would give rise to volume expansion, leading to the formation of microcracks inside the mortar [37]. While for specimens premixed Ca^2+^ (E-ISG, E-ISMG, N-ISG and N-ISMG), the mass loss induced by the dehydration of ettringite and C-S-H gel is clearly higher in the range of 90–120 °C. It can be inferred that more ettringite and C-S-H gel are generated in mortars with premixed Ca^2+^. The C-S-H gel, as one of the main products of cement hydration, plays an important role in maintaining the stability of the mortar structure. Therefore, the strength and porosity of mortar would be enhanced since Ca^2+^ promotes the formation of C-S-H gel.

## 4. Discussion

This paper studied the degradation mechanisms of cast-in-situ mortar that suffered internal–external multiple ion attacks. At present, most of the literature has mainly researched the degradation mechanisms of external multiple ion attacks on concrete or mortar after 28 days or 14 days of standard curing [7,21,38,39,40]. Cast-in-situ structures are widely used in all kinds of engineering construction. Such a situation brings great differences in degradation mechanisms between cast-in-situ structures and precast structures. In addition, researchers have always focused on external multiple ion attacks rather than internal ion attacks. In fact, the damage induced by internal ions attack (SO_4_^2−^, Mg^2+^, Ca^2+^) cannot be ignored in civil engineering as well.

### 4.1. ESA-ISA-IMA

ISA or ISA-IMA would exist during the mixing process until the interior aggressive ions are entirely consumed. The internal aggressive ion-induced attack accelerates the early degradation of cast-in-situ mortars induced by ESA. Multiple corrosion products generated by ISA and IMA negatively affect inherent pore structures, leading to varying degrees of damage.

For cast-in-situ structures that suffered CSA, reactions between sulfate ions and cement composites would occur in both mixing and hardening processes (as proved by XRD and TG/DTG analysis in Figure 9 and Figure 10) [3]. Corrosion reactions inside mortar persistently proceed until sulfate ions are fully consumed. Fine cracks caused by expansive corrosion products accumulate inside mortar during the early stage, as can be seen in Figure 7a. This crack system would be continuously expanded with the accumulation of expansive corrosion products and cause severe degradation, which would also increase the percentage of relatively large pores (shown in Figure 5) [13]. Furthermore, microcracks contribute to the penetration and accumulation speed of external sulfates (shown in Figure 6), generating more expansion degradation products accumulated in the surface layer of mortar. Early degradation and micro-expansion induced by CSA would lead to peeling, as well as the generation of the crack system over time, negatively affecting the strength development of specimens. Soon, complete failure of cast-in-situ structures would occur after concrete or mortar loses most of its strength.

For cast-in-situ structures that suffer ESA-ISA-IMA, deterioration is more severe due to more complicated degradation mechanisms. There are more magnesium-induced products (including Mg(OH)_2_ and non-cementitious M-S-H, as shown in the results of XRD and TG/DTG analysis) besides ettringite and gypsum induced by ISA [16]. Interior tensile stress caused by magnesium attack is generated and loaded into internal mortar, causing the destruction of cast-in-situ structures [41]. The corrosion products generated by ISA-IMA enlarge the initial porosity of the mortar, which negatively affects the development of concrete or mortar strength (as indicated by the results of porosity and strength tests). In this regard, one can conclude that ISA-IMA has negative effects on the durability of concrete or mortar structures.

In addition, it is noteworthy that there are abundant flake-like magnesium hydrates near or growing together with ettringite crystals, as shown in Figure 7b,c. It blocks the diffusion paths of external sulfates and retards the expansion of microcracks, enhancing the durability of mortar anti-sulfate attacks [16]. However, it cannot sufficiently resist the degradation tendency induced by IMA. This hypothesis can also be observed and inferred from the curves of compressive strength in Figure 4.

### 4.2. Effect of Ca^2+^ on ESA-ISA-IMA

For cast-in-situ structures that suffered ISA or ISA-IMA, internal SO_4_^2−^ and Mg^2+^ would react with Ca(OH)_2_ until these corrosion ions are entirely consumed, forming both sulfate- and magnesium-induced corrosion products (shown in Figure 7, Figure 8, Figure 9 and Figure 10). When Ca(OH)_2_ in the pore solution is insufficient to react with SO_4_^2−^ and Mg^2+^, calcium is released from the C-S-H gel phase. Eventually, the decomposition of the C-S-H gel causes the degradation of cement or mortar [6,31]. After premixing extra calcium salts, sufficient Ca^2+^ is provided to react with SO_4_^2−^ and Mg^2+^. This decreases the decalcification of C-S-H and preserves the integrity of these gels [6,37,40]. Besides, Ca^2+^ would also facilitate the generation of C-S-H gel and cotton-shaped gel (as shown in Figure 7c,d), forming a special frame structure. From the obtained results of specimen strength, porosity and sulfate concentration in mortar, one can conclude that Ca^2+^ would contribute to the strength development and structure stability of mortar. The improvement of pore structure retards the diffusion of external sulfates and enhances the durability of cast-in-situ mortars against ESA.

The early hydrate reaction of cement is facilitated by excessive Ca^2+^ premixed in cast-in-situ mortars. Mechanical properties (both flexural strength and compressive strength) and porosity would be improved since Ca^2+^ makes the microstructure of mortar denser. Therefore, Ca^2+^ would improve the mechanical properties and pore structure of mortars suffering from both ESA and ISA-IMA.

## 5. Conclusions

This experiment studied the effect of Ca^2+^ on the early degradation of cast-in-situ mortars suffering from ESA-ISA-IMA, as well as the degradation mechanisms of cast-in-situ mortars induced by ESA-ISA-IMA. The physical and mechanical properties, porosity and microstructures of the mortar specimens were determined and discussed in detail. The conclusions are as follows:

(1) ISA and ISA-IMA negatively influence both pore structures and the early strength development of cast-in-situ mortars. Early expansion and microcracks induced by multiple internal–external combined attacks would accelerate the process of degradation induced by ESA.
(2) Flake-like magnesium hydrates hinder the expansion of microcracks and the penetration of external sulfate ions. However, it would not be enough to neutralize the negative effects induced by IMA.
(3) Premixed excessive Ca^2+^ would retard the degradation triggered by CSA or ESA-ISA-IMA and improves the durability of cast-in-situ structures against sulfate-rich environments. Sufficient Ca^2+^ would improve the porosity and strength of mortar during the early hydration process.

## Figures and Tables

**Table 1:** Chemical composition of cement.

**Composition**	CaO	SiO_2_	Al_2_O_3_	Fe_2_O_3_	SO_3_	MgO	Cl	Na_2_O	TiO_2_	K_2_O
**Content (%)**	60.71	20.45	4.14	2.85	2.73	1.63	0.027	0.702	0.339	0.44

**Table 2:** Mix proportion.

W/C	Water (kg/m^3^)	Cement (kg/m^3^)	Standard Sand (kg/m^3^)
0.5	1	2	3

Note: W/C is the water-cement ratio.

**Table 3:** Sizes and purposes of the samples.

Sample	Size (cm)	Purpose
A	4 × 4 × 16	Flexural strength, sulfate concentration, weight and size changes, appearance
B	5 × 5 × 5	Compressive strength, porosity, SEM-EDS, XRD and TG/DTG analysis

**Table 4:** Information on specimens and corrosion conditions.

Solutions		Internal Attack		
No External Sulfate	External Sulfate Solution	SO_4_^2−^	Mg^2+^	Ca^2+^
N-CK	E-CK	0	0	0
N-IS	E-IS	3%	0	0
N-ISM	E-ISM	3%	3%	0
N-ISG	E-ISG	3%	0	3%
N-ISMG	E-ISMG	3%	3%	3%

Note: “N” represents samples immersed in distilled water; “E” represents samples exposed to sulfate solution; “I” represents internal ion attacks.

## Data Availability

The raw/processed data required to reproduce these findings cannot be shared at this time as the data also forms part of an ongoing study.
